# Modelling the failure precursor mechanism of lamellar fibrous tissues, example of the annulus fibrosus

**DOI:** 10.1016/j.jmbbm.2016.06.030

**Published:** 2016-10

**Authors:** Marlène Mengoni, Alison C. Jones, Ruth K. Wilcox

**Affiliations:** a Institute of Medical and Biological Engineering, School of Mechanical Engineering, University of Leeds, Leeds LS2 9JT, UK; b Institute of Medical and Biological Engineering, University of Leeds, UK

**Keywords:** Inter-lamellar behaviour, Fibrous tissues, Damage, Failure, Interface

## Abstract

The aims of this study were to assess the damage and failure strengths of lamellar fibrous tissues, such as the anterior annulus fibrosus (AF), and to develop a mathematical model of damage propagation of the lamellae and inter-lamellar connections. This level of modelling is needed to accurately predict the effect of damage and failure induced by trauma or clinical interventions. 26 ovine anterior AF cuboid specimens from 11 lumbar intervertebral discs were tested in radial tension and mechanical parameters defining damage and failure were extracted from the in-vitro data. Equivalent 1D analytical models were developed to represent the specimen strength and the damage propagation, accounting for the specimen dimensions and number of lamellae. Model parameters were calibrated on the in-vitro data. Similar to stiffness values reported for other orientations, the outer annulus was found stronger than the inner annulus in the radial direction, with failure at higher stress values. The inner annulus failed more progressively, showing macroscopic failure at a higher strain value. The 1D analytical model of damage showed that lamellar damage is predominant in the failure mechanism of the AF. The analytical model of the connections between lamellae allowed us to represent separately damage processes in the lamellae and the inter-lamellar connections, which cannot be experimentally tested individually.

## 1 Introduction

A number of surgical techniques in the intervertebral disc, such as needle puncture during nucleus replacement, discography, or simple annulus suturing, can cause direct or progressive damage leading to degeneration (Carragee et al., 2009, Michalek et al., 2010, Nassr et al., 2009). Modelling the failure mechanisms of the inter-vertebral disc can provide a better understanding of those effects beyond direct observation. While disc degeneration is caused by both mechanical and biochemical factors (Adams and Roughley, 2006, Alini et al., 2008), damage, failure, and fracture during or directly following an intervention can have a purely mechanical origin. In order to model direct failure of the disc, the tissue damage and fracture strength need to be captured in the mathematical description of the tissue behaviour. While the elastic behaviour of the annulus fibrosus has been reported to vary across radial and circumferential locations (Elliott and Setton, 2000, Holzapfel et al., 2005), there is a lack of in-vitro evidence regarding non-elastic behaviour such as damage and fracture strength. In particular, the contribution of the inter-lamellar connections (Pezowicz et al., 2005, Pezowicz et al., 2006, Schollum et al., 2008) on the strength of the annulus fibrosus has not been quantified. The strength of similar connective tissues is also poorly studied in other lamellar soft tissues, such as blood vessels, or in connections between articular soft tissues such as the labro-chondral junction.

The aims of this study were to assess the damage and failure strength of the anterior annulus fibrosus (AF) under radial tension, and to develop a mathematical model of damage propagation of the lamellae and inter-lamellar connections in that direction. The development of the proposed theoretical model of damage propagation allowed us to isolate the contribution of lamellar and inter-lamellar constituents, which cannot be experimentally tested individually, in the damage mechanisms of the AF. This work provides data on the non-fibrillar matrix damage properties through the specific loading mode as well as a first step towards the inclusion of damage in *in-silico* models of lamellar fibrous tissues.

## 2 Materials and methods

The ovine disc was chosen for this study because it is commonly used as an in-vivo model (Hegewald et al., 2015, Reitmaier et al., 2012) and due to its biochemical and structural similarities to the human disc (Alini et al., 2008, Wilke et al., 1997).

### 2.1 Specimen preparation and mechanical testing

Specimen preparation and testing methods have been reported in a previous study (Mengoni et al., 2015) and are summarised here.

**Fig. 1:**
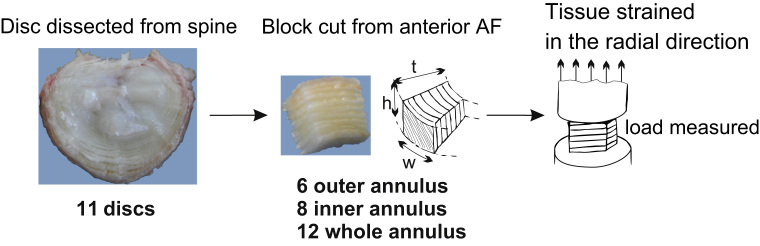
specimen preparation and testing (adapted from Mengoni et al. (2015)), showing an example of an outer annulus specimen. Dimensions of the specimen are referred as disc height, *h*, specimen thickness, *t*, and specimen width, *w* (see Table 1).

Lumbar intervertebral discs were extracted from frozen mature ovine spines stored at −20 °C in a plastic wrap and were classified as having a low level of degeneration by two independent reviewers (grade I or II following Thompson׳s scale (Thompson et al., 1990)). Anterior annulus intervertebral disc samples (*N*=26) were carefully extracted from a total of 11 discs and prepared as rectangular parallelepiped specimens (see Fig. 1). They were divided into three groups depending on their radial location in the anterior annulus: outer annulus (*N*=6), inner annulus (*N*=8) and samples across the whole annulus thickness (*N*=12). The dimensions of each specimen were measured with a caliper and the number of lamellae per sample was recorded by visual inspection. After dissection and prior to tensile testing, all specimens were floated in 0.10 M saline solution and tested within an hour of the disc extraction from the frozen spine. For each specimen, the outer-most layer was glued onto the end of an axial cylinder fitting into a materials testing machine (Instron 5543, Instron Corp., Norwood, MA, fitted with a 50 N static load cell). The inner-most layer was glued to the bottom plate of the testing machine in such a way that the specimen was compressed by a maximum of 1% to achieve full contact. Tensile radial tests were performed at a crosshead speed of 1 mm/min while recording the load. The specimens were extended between an apparent tensile strain of 20% and macroscopic failure. Specimens that were not tested to failure were plunged for 2.5 h in a bath of 10% neutral buffered formalin while maintained at a constant strain level. As a result, specimen fixation under tension was obtained. The fixed specimens were then sectioned (30 μm thickness) and imaged by differential interference contrast (DIC) microscopy.

### 2.2 Definition of experimental mechanical data

**Fig. 2:**
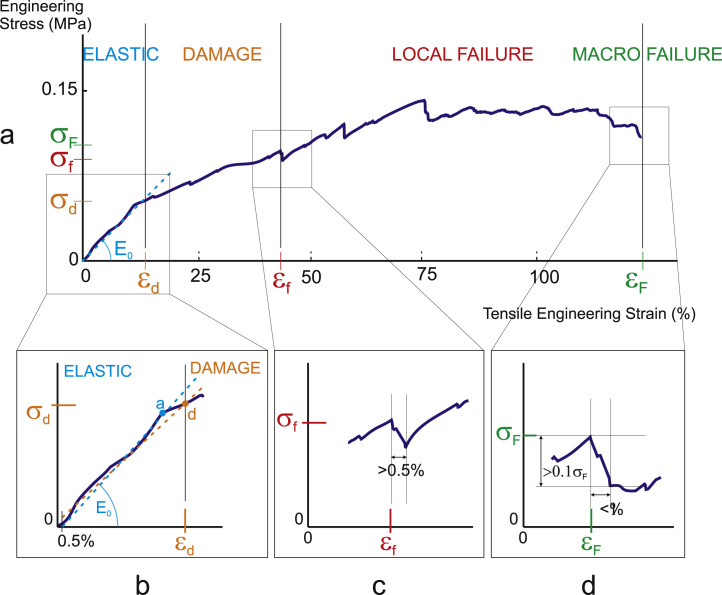
typical stress/strain data with (a) definition of the in-vitro mechanical parameters; (b) difference between the linear fit used in the definition of the specimen apparent stiffness (from 0.5% to a, defined from the change of curvature) and the apparent occurrence of damage (from 0.5% to d, defined from the change of RMS error for a linear fit); (c) definition of local failure; (d) definition of macroscopic failure.

Experimental apparent mechanical data (see Fig. 2(a)) was derived from the load-extension curves. Apparent stress and strain were computed by normalising the load by the apparent surface area, and the extension by the initial sample height. All data was resampled at 0.05% strain steps. The radial apparent elastic modulus, *E*_0_, was derived for each sample as the slope of a linear fit between 0.5% strain and the strain value at which the curvature of the apparent stress/strain data deviated from zero (point a in Fig. 2(b)). The first 0.5% tensile strain was discarded to suppress boundary contact effects. Given the non-perfectly linear behaviour of the small strain behaviour, deviation from zero was defined as a value above a threshold set at 50 MPa. Occurrence of apparent damage was defined as the set of apparent stress/strain values ($\sigma_{d},\varepsilon_{d}$) for which the non-linearity has increased by 20%, i.e. for which the RMS error of a linear fit from 0.5% strain was higher by 20% than the RMS error of the linear fit defining the elastic modulus (see point d in Fig. 2(b)). Initial local failure was defined as the set of stress/strain values ($\sigma_{f},\varepsilon_{f}$) at the start of loss of load bearing capacity, ie at the start of a negative slope that occurred over a strain range of more than 0.5% (see Fig. 2(c)). Subsequent local failure events were defined similarly. Macroscopic failure stress/strain values ($\sigma_{F},\varepsilon_{F}$) were defined as the points of substantial loss of load, ie the points at which the stress dropped by more than 10% over less than a 1% increment in strain (see Fig. 2(d)). Failure events were corroborated with image analysis of the sections of tissue fixed at different apparent strains values.

The measured mechanical parameters were compared between groups and within each group. Given the low number of samples and the non-Gaussian nature of the data, the values were compared using a Kruskal-Wallis test and the corresponding paired comparisons with Mann-Whitney tests using statistical software (R.3.1.1, R foundation for statistical computing). Bonferonni corrections were applied for each stress and strain values to account for the number of between-groups and within-groups paired comparisons. The non-adjusted criterion for statistical significance was set at *p*<0.05.

### 2.3 Analytical modelling of damage

A 1D variable-stiffness spring model was designed to replicate the damage part of the experimental data, i.e. for strain values in a range from occurrence of damage, $\varepsilon_{d}$, to initial local failure, $\varepsilon_{f}$. Springs were assembled in series representing either the lamellae or the inter-lamellar connections, with a number of lamellar springs equal to the measured number of lamellae for each sample. All lamellar springs were assumed to exhibit the same strain dependent stiffness, so were the inter-lamellar connection springs.

**Fig. 3:**
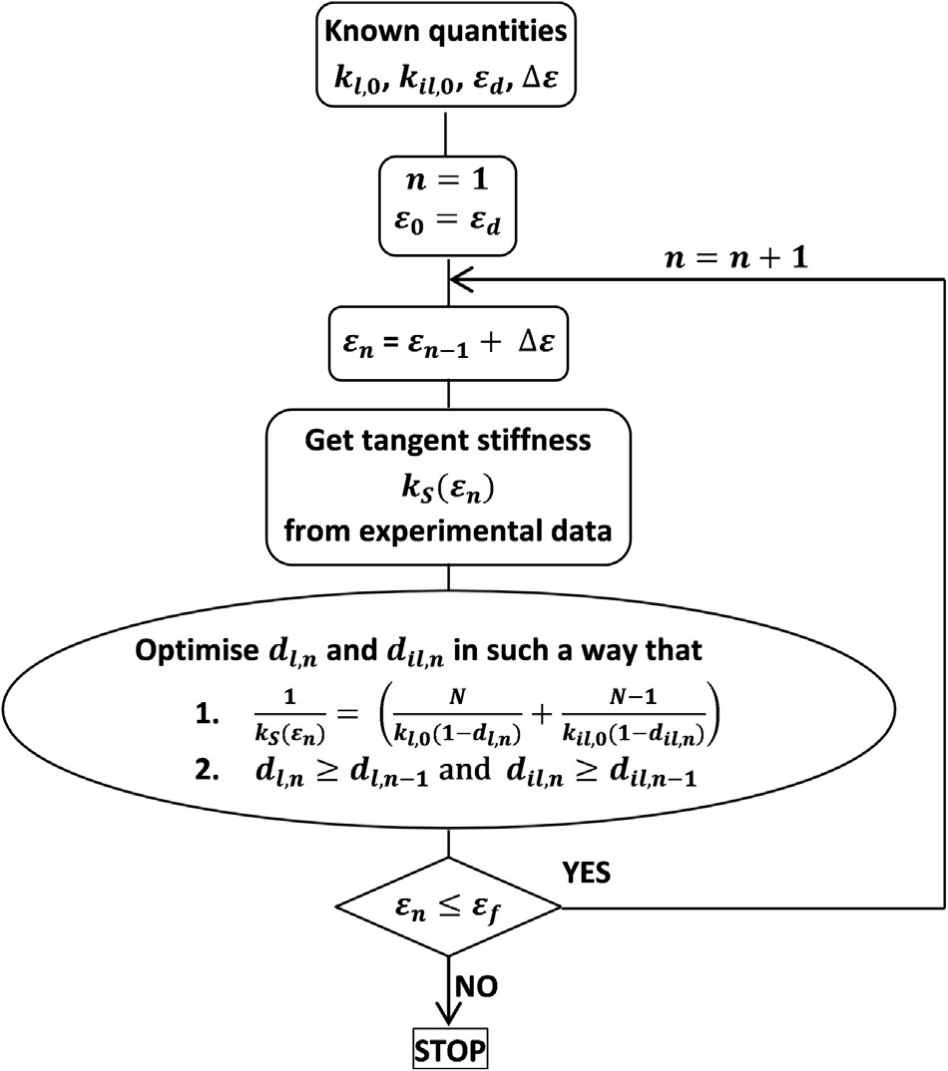
flowchart of the derivation of damage values for each group of specimens, where *N* is the number of lamellae.

The initial stiffness values of the lamellar and inter-lamellar springs, $k_{l,0}$ and $k_{\mathrm{il},0}$, were derived from an elastic model (Mengoni et al., 2015). The damaged springs were modelled in line with the continuum damage mechanics theory (Lemaitre and Desmorat, 2005). Damage is measured by an internal variable d related to the effective density of cracks in the tissue. The damage variable is normalised in such a way that the value d=0 corresponds to the undamaged tissue while the value d=1 represents the local failure of the tissue. Using the strain equivalence approach of damage, the stiffness is reduced with increasing strains according to Eq. (1):

(1) $$k_{l/\mathrm{il}}\left(\varepsilon \right)=\{\begin{array}{l} k_{l/\mathrm{il},0}\;\varepsilon \leq \varepsilon_{d}\\ \left(1-d_{l/\mathrm{il}}\left(\varepsilon \right)\right)k_{l/\mathrm{il},0}\;\varepsilon >\varepsilon_{d} \end{array}$$

where $\varepsilon_{d}$ is the strain value at the occurrence of damage, l/il stands either for lamellar or inter-lamellar springs, and $d_{l/\mathrm{il}}\left(\varepsilon_{d}\right)=0$. The damage values of the lamellar and inter-lamellar springs at each strain step, $d_{l/\mathrm{il}}\left(\varepsilon \right),$ were computed for each group of specimens (see Fig. 3). An optimisation process to minimise the difference in a least-square sense between in-vitro secant stiffness, $k_{S}\left(\varepsilon \right),$ and analytical equivalent spring stiffness was developed using the Python implementation of the Levenberg-Marquardt algorithm (Levenberg, 1944, Marquardt, 1963) in LMFIT-py (http://lmfit.github.io/lmfit-py/). In order to respect thermodynamic principles, the damage $d_{l/\mathrm{il}}$ at a given strain value ε was computed in such a way that it could not decrease from the corresponding damage value computed at a lower strain value. Given the annulus model was composed of damaged springs in series, representative of either the lamellae or the inter-lamellar connections, damage variation was derived for both of these groups as a whole and not for each lamella or connection separately.

Finally, to derive a damage evolution law, exponential decay functions (Eq. (2)) or sigmoid functions (Eq. (3)) were fitted to the obtained damage/strain curves using a curve fitting toolbox (MATLAB® R2014b, The Mathworks Inc., Natick, MA, US):

(2) $$d_{l/\mathrm{il}}\left(\varepsilon \right)=a\left(1-e^{-b(\varepsilon -\varepsilon_{d})}\right)$$

or

(3) $$d_{l/\mathrm{il}}\left(\varepsilon \right)=\frac{m}{1+e^{-n\left(\varepsilon -\left(\varepsilon_{d}+\varepsilon_{0}\right)\right)}}$$

with ${a,b\;\text{or}\;m,n,\;\text{and}\;\epsilon }_{0}$ calibrated on the damage/strain curves. Sigmoid functions were used where fitting exponential decay functions led to *R*^2^ values below 0.9.

## 3 Results

The data associated with this paper (geometrical dimensions, mechanical testing, DIC images) as well as the procedures to define mechanical parameters are openly available from the University of Leeds Data Repository (Mengoni and Wilcox, 2016).

### 3.1 Experimental results

**Fig. 4:**
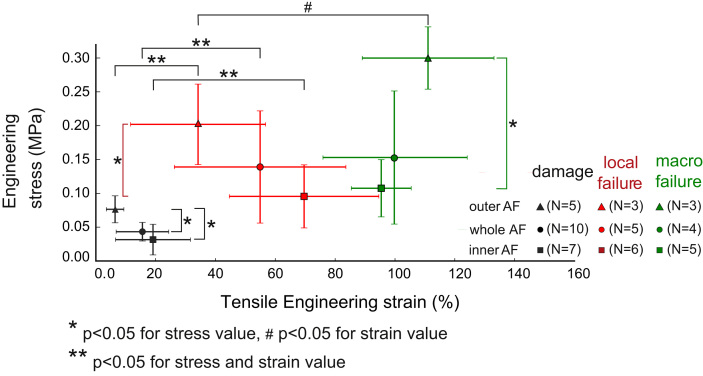
experimental stress/strain values defining occurrence of damage, first local failure and macroscopic failure for the outer annulus samples, the inner annulus samples and the samples across the annulus thickness.

Mean values of the specimen geometrical dimensions in each group are presented in Table 1. As reported previously (Mengoni et al., 2015), the dimensions of a subset of 5 samples were measured before and after being floated in saline. No change in dimensions was observed within the measurement precision. Measured mechanical parameters are presented in Fig. 4, reporting all significant differences, and the key findings are discussed here after.

Within each group, significant differences were found between the stress and strain values at the occurrence of damage ($\sigma_{d},\varepsilon_{d}$) and at initial local failure ($\sigma_{f},\varepsilon_{f}$). However, no significant difference was observed between the stress at the occurrence of initial failure ($\sigma_{f}$) and at macroscopic failure ($\sigma_{F}$).

The outer annulus group exhibited a higher stress level at occurrence of damage, at initial failure and at macroscopic failure than the inner annulus group. The strain range during which failure progressed from initial local failure to macroscopic failure was higher for the outer annulus group than for the inner annulus group, with a higher number of local failure events in the outer annulus group. No significant differences between groups were found in the strain values of all failure data.

**Fig. 5:**
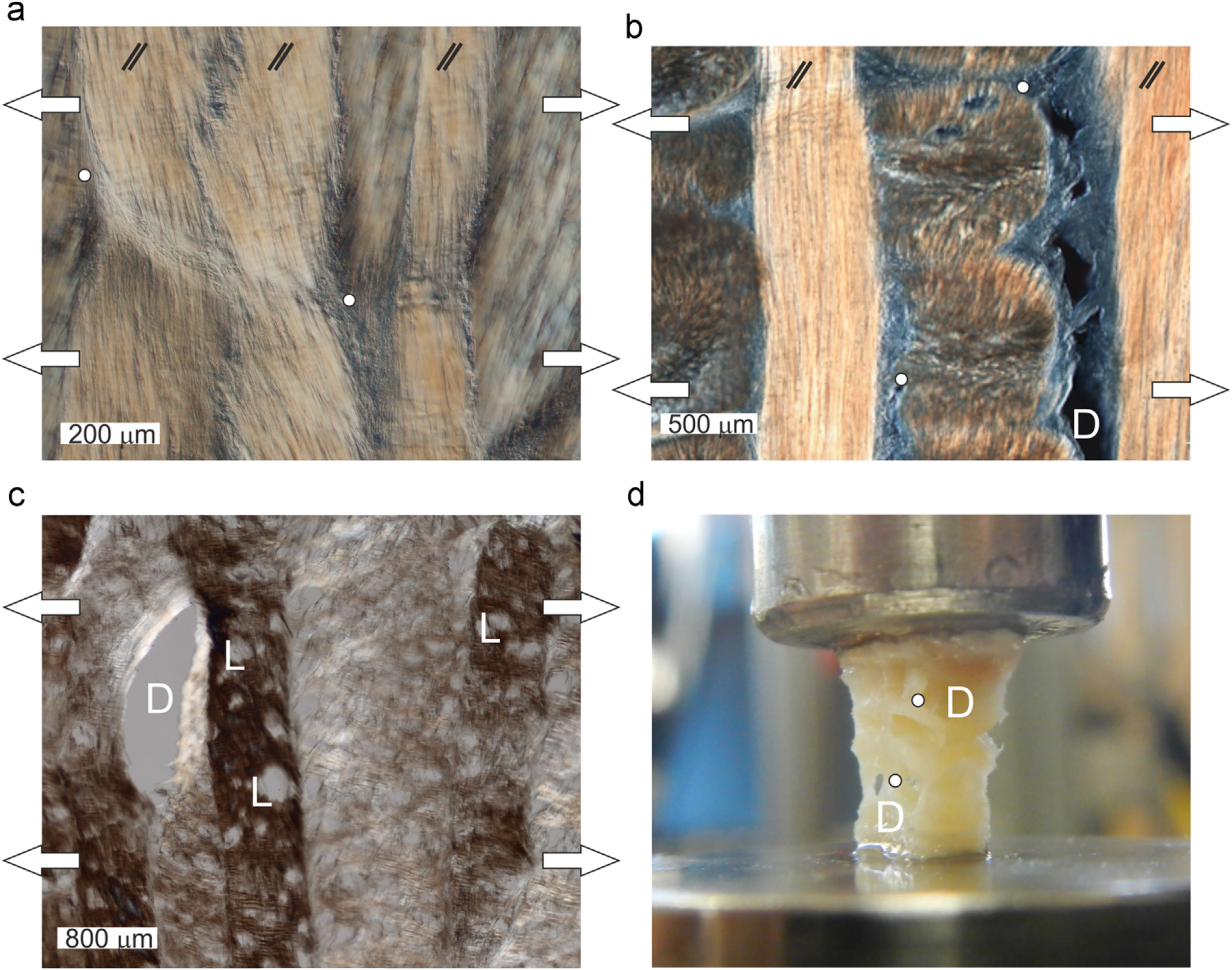
Differential Interference Contrast microscopy image of a slice of annulus fibrosus stretched radially to (a) 20%; (b) 50% and (c) 100%. (a) and (b) 10× magnification slices cut in-plane with fibres orientation in every other lamellae (marked as //); (c) 4× magnification of a slice cut in the transverse plane. (d) Photograph of a specimen before macroscopic failure. White arrows represent direction of stretch. Inter-lamellar bridges at the edge of local failure are highlighted with a circle. D indicates the location of clear delamination and L indicates lamellar failure. Further images available as supplementary data (Mengoni and Wilcox, 2016).

Local damage and micro-fractures can be observed at strain level from 20% onwards, with increasing micro-damage of the structural components as strain increase. The use of DIC microscopy on samples prepared so that alternate lamellae are cut in plane allowed visualisation of the fracture within inter-lamellar connections. The commonly observed microstructural deformation modes are depicted in Fig. 5. The presence of bridges and inter-lamellar fibre network is clearly depicted on the in-plane sections (Fig. 5(a) and (b)) while the transverse section (Fig. 5(c)) shows the distributed local failure of the specimen. Where no bridges are present, the inter-lamellar elastin network cannot sustain the load and fails at the microstructural level, facilitating delamination (denoted by D on Fig. 5). The presence of bridges however reinforces the interface between lamellae (denoted by a white circles on Fig. 5). Before macroscopic failure (Fig. 5(c)), the annulus section withstands load mainly through the secondary bridge structure of the tissue as the lamellar tissue is failing (denoted by L on Fig. 5). Clear local macroscopic delamination was observed before failure (Fig. 5(d)).

### 3.2 Damage modelling results

**Fig. 6:**
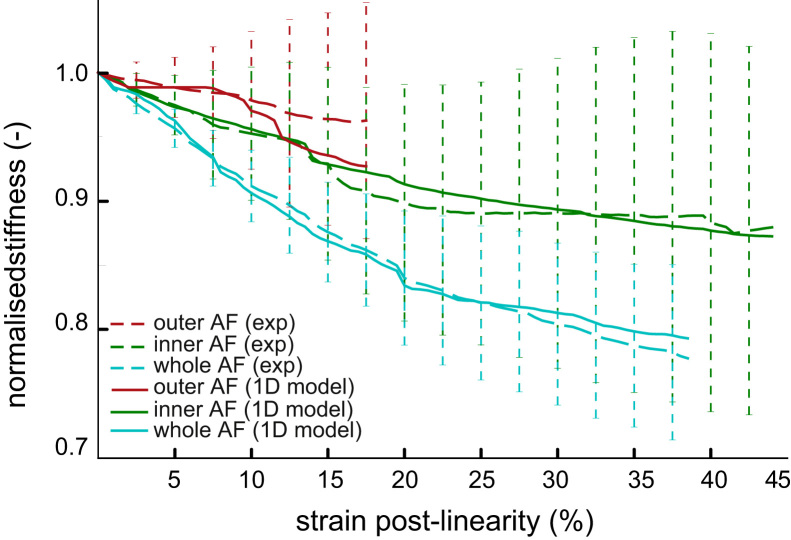
1D damage model fit on the stiffness/strain data (mean+/−std) for all three groups.

The optimisation converged to damage values reproducing the sample stiffness with a specimen RMS error of maximum 10% (Fig. 6), with a better agreement for the inner annulus and whole annulus groups than for the outer annulus one.

**Fig. 7:**
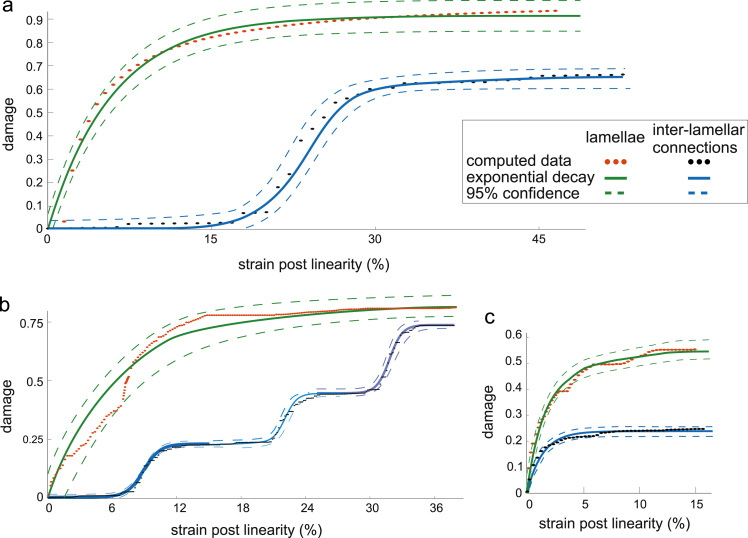
computed (1D) damage variation for the lamellae and inter-lamellar connections and fitted exponential functions, (a) inner annulus group, (b) whole annulus group, (c) outer annulus group.

At a given strain level, the predicted damage of the lamellae was always higher than that of the inter-lamellar connections. An exponential law was fitted on each damage evolution data with the strain as independent variable with correlation values reported in Table 2 and Fig. 7. The inter-lamellar connections of the whole annulus group were fitted with three consecutive sigmoids over the strain range and not a single one (see Fig. 7(b)).

## 4 Discussion

The experimental data presented here suggests that, in radial loading, the annulus has an almost linear behaviour up to about 8 to 20% strain depending on location, followed by an apparent stiffness decrease until the first instance of local failure. Several local failures follow at similar stress levels before macroscopic failure. The stress level at the occurrence of damage and failure depends on the radial location of the samples. The nearly linear behaviour at low strain values and sub-linear behaviour at higher strains can be explained by the loading conditions. In radial loading, the fibres of the lamellae do not bear any load. Only the non-fibrillar matrix of the lamellae and the lamellar interconnections sustain the load. A similar linear range has been previously reported (Cortes and Elliott, 2012, Fujita et al., 1997, Stewart et al., 2016) for the non-fibrillar matrix. The outer annulus showed a more progressive failure mechanism than the inner annulus. This can be characterised in terms of standard mechanics of material terminology as the outer annulus being stiffer, stronger and more ductile than the inner annulus.

The analytical model of the connections between lamellae allowed representation separately of the damage processes in the lamellae and the inter-lamellar connections. The model shows that lamellar damage is predominant in the damage mechanism of the samples. This result is in good agreement with the extensive qualitative data analysed in Pezowicz et al. (2006). Indeed it was reported from image analysis that inter-lamellar connections such as cross-bridges contributed to the radial strength of outer annulus samples extracted from caudal bovine discs with a highly hierarchical splitting at high strain values; the complex splitting and sub-splitting with local fragmentation prevents the connections from reaching full failure. Similar strong inter-lamellar connections were also observed in the present ovine study.

The theoretical damage, $d_{l/\mathrm{il}},$ considered in this work is a reduction in apparent stiffness of the lamellae and inter-lamellar connections. It does not directly reflect the amount of micro-fractures or physical damage of the inter-lamellar connections. The strong physical anchorage of the inter-lamellar elements and their hierarchical splitting mode insure they contribute to the overall radial stiffness even when physically highly damaged. This damage, while being physically higher than that in the lamellae (Pezowicz et al., 2005, Pezowicz et al., 2006), does not contribute as much in the total loss of stiffness in radial tension. The mathematical value of damage in the model is therefore higher for the lamellar elements than for the inter-lamellar ones. This is also in agreement with failure interpretation (Pezowicz et al., 2006) suggesting that intra-lamellar failure occurs before full inter-lamellar connection failure.

It should be noted however that the developed mathematical description of damage in the radial direction is a continuous representation of the connections between lamellae while the inter-lamellar connections have been reported to be localised entities (Schollum et al., 2008). The exact location of failure onset would not be predicted with such a mathematical description, only the strength and varying stiffness with increasing strain is represented in the interface continuous damage approach proposed here. The difference in behaviour between lamellae or between connections is not captured by the mathematical model as all similar entities are assumed to contribute in the same way to the apparent radial stiffness. Accounting for the difference in thickness or modulus of each entities could be done with a similar approach but requires data not available in this study.

The results produced in this study are valid when assessing the strength of an in-vitro annulus specimen. Results cannot be directly extrapolated for an in-situ specimen. In particular, they are not representative of the strength of the annulus in an area close to the endplates of the intervertebral disc where it has been shown that annulus and nucleus fibres integrate into the fibrous cartilaginous endplate (Rodrigues et al., 2015, Wade et al., 2011). It is likely that this intricate anchorage of the intervertebral disc into the vertebrae contributes to the absolute strength of the disc in-situ.

The methods proposed in this work assessed the damage variation before the first occurrence of failure in radial tension. This loading direction was selected partly in order to understand the non-fibrillar matrix behaviour as the lamellar fibres do not bear any load in the radial direction. The matrix is more likely to have an isotropic behaviour than the lamellar fibres or the inter-lamellar connections, so the behaviour derived in this specific loading scenario is likely to be valid in other loading directions. While the results indicated that first failure will occur at strain levels that may not be physiologically relevant in the radial direction tested, higher strains occur in other loading directions and the damage properties derived here for the non-fibrillar matrix become more physiologically relevant. Damage in radial tension is unlikely to be the main failure mechanism of the annulus fibrosus in clinical interventions. However, the developed methods can now be applied in the circumferential and axial directions, in tension and compression, to produce a full 3D picture of damage propagation in lamellar tissues. This is an essential step in enabling modelling of the structural damage caused by interventions.

Finally, ovine and human discs have somewhat different morphologies and no direct quantitative comparison is offered at the full disc level. However, the sub-structural components (inter-lamellar bridges and elastin matrix) are present in both types of tissues. The purely structural mechanisms to damage and failure are thus probably similar, especially in the radial direction where the fibres do not bear load and the difference in fibre density between both types of tissues is less likely to have a large influence. The methods proposed in this work should thus be easily translated to human tissues. It is however possible that they do not apply to older, degenerated, tissue where there are likely to be discontinuities in the tissue and the contributions of the different entities may be more variable.

The methods developed in this work are generic and apply to any lamellar material where the sub-components gets damaged in different ways but cannot be tested individually. This is true in particular in vessels such as blood vessels (Gasser et al., 2010) or the trachea (Baiguera et al., 2012) and the oesophagus (Sommer et al., 2013). More-over, extension to interfaces such as the labro-chondral junction (Ferguson et al., 2001) would enable understanding of delamination processes in these soft-tissues.

The results reported in this work demonstrated that the precursor of failure in the annulus in the radial direction can be adequately modelled using continuum damage theory. Such material models, combined with further characterisation in circumferential and radial directions, can feed into 3D finite element models of the intervertebral disc and be used to predict outcomes of clinical interventions involving needle puncture, tissue dissection, or simple suturing.

## Figures and Tables

**Table 1:** Specimen geometrical dimensions (mean+/−std, in mm), see Fig. 1 for labelled schematic.

	Disc height *h*	Specimen thickness *t*	Specimen width *w*
Inner annulus	3.95+/−0.6	3.59+/−0.4	6.42+/−1.7
Outer annulus	4.13+/−1.0	3.42+/−0.8	6.88+/−0.8
Whole annulus	3.67+/−0.4	4.52+/−0.9	5.94+/−1.0

**Table 2:** Exponential law fitting the damage evolution with strain.

	Exponential decay Eq. (2)		Sigmoid function Eq. (3)	
	RMS error	*R*^2^	RMS error	*R*^2^
Inner annulus lamellae	0.032	0.97		
Inner annulus inter-lamellar connections	–	<0.9	0.0095	0.98
Outer annulus lamellae	0.027	0.96		
Outer annulus inter-lamellar connections	0.0086	0.98		
Whole annulus lamellae	0.0021	0.92		
Whole annulus inter-lamellar connections	–	<0.9	0.045	0.97
